# Multidisciplinary Rehabilitation is Efficacious and Induces Neural Plasticity in Multiple Sclerosis even when Complicated by Progressive Multifocal Leukoencephalopathy

**DOI:** 10.3389/fneur.2017.00491

**Published:** 2017-09-19

**Authors:** Elisabetta Groppo, Francesca Baglio, Davide Cattaneo, Eleonora Tavazzi, Niels Bergsland, Sonia Di Tella, Riccardo Parelli, Ilaria Carpinella, Cristina Grosso, Ruggero Capra, Marco Rovaris

**Affiliations:** ^1^IRCCS Fondazione Don Gnocchi ONLUS, Milan, Italy; ^2^Buffalo Neuroimaging Analysis Center, Department of Neurology, University at Buffalo SUNY, Buffalo, NY, United States; ^3^ASST Spedali Civili of Brescia, MS Regional Center, Montichiari, Italy

**Keywords:** multidisciplinary rehabilitation, multiple sclerosis, progressive multifocal leukoencephalopathy, functional magnetic resonance imaging, neuroplasticity

## Abstract

A 48-year-old woman with multiple sclerosis (MS), treated with natalizumab for more than one year without clinical and magnetic resonance imaging (MRI) signs of disease activity, was diagnosed with definite progressive multifocal leukoencephalopathy (PML). She presented with subacute motor deficit of the right upper limb (UL), followed by involvement of the homolateral leg and urinary urgency. The patient was treated with steroids and plasma exchange. On follow-up MRI scans, the PML lesion remained stable and no MS rebounds were observed, but the patient complained of a progressive worsening of the right UL motor impairment, becoming dependent in most activities of daily living. A cycle of multidisciplinary rehabilitation (MDR) was then started, including daily sessions of UL robot therapy and occupational therapy. Functional MRI (fMRI) was acquired before and at the end of the MDR cycle using a motor task which consisted of 2 runs: in one run the patient was asked to observe while the second one consisted of hand grasping movements. At the end of the rehabilitation period, both the velocity and the smoothness of arm trajectories during robot-based reaching movements were significantly improved. After MDR, compared with baseline, fMRI showed significantly increased functional activation within the sensory-motor network in the active, motor task, while no significant differences were found in the observational task. MDR in MS, including robot-assisted UL training, seems to be clinically efficacious and to have a significant impact on brain functional reorganization on a short-term, even in the presence of superimposed tissue damage provoked by PML.

## Introduction

Multiple sclerosis is a chronic disease with both inflammatory and degenerative pathological features, leading to disability and functional impairment (1). In the last 20 years, several pharmacological therapies proved to be effective in favorably modifying the course of MS, by reducing the frequency and severity of disease relapses and the risk of disability accrual (1). Among these treatments, natalizumab, a monoclonal antibody against the alpha-4 chain of VLA-4, that mediates cell migration and infiltration in the central nervous system, is approved as monotherapy for rapidly worsening relapsing-remitting MS (2). Its use, however, has been associated with progressive multifocal leukoencephalopathy (PML). Although strategies have been developed for reducing its risk (3), the occurrence of PML in natalizumab-treated MS patients can lead to death or irreversible disability in more than one third of the cases (2, 4).

The efficacy of multidisciplinary rehabilitation (MDR) in promoting functional recovery and increasing quality of life in MS patients has been demonstrated by several studies (5). The possible biological basis for such a recovery seems to include both the stimulation of neurotrophic factors (6) and the enhancement of functional and structural brain plasticity (7). Neural plasticity is defined as the ability of the nervous system to respond to intrinsic or extrinsic stimuli by reorganizing its structure, function, and connections (8). These phenomena can be investigated *in vivo* by means of structural and functional (f) MRI-based techniques (9). In MS patients, functional MRI (fMRI) can detect patterns of cortical activation in response to motor or cognitive tasks and monitor their changes over time, which differ from those of healthy subjects (10, 11). Published studies indicate that, following rehabilitation interventions, fMRI patterns can change in MS patients and reflect adaptive reorganization to damage in parallel with functional improvement (7–9, 11).

To the best of our knowledge, little is known about the dynamics of neural plasticity in MS patients experiencing superimposed damage provoked by PML. This report describes the outcomes of MDR intervention, including upper limb (UL) robotic treatment, in an MS patient with PML. Our aim was to assess whether rehabilitation can still induce functional recovery by promoting neural plasticity despite the combination of the two diseases.

## Case Presentation

**Figure 1:**
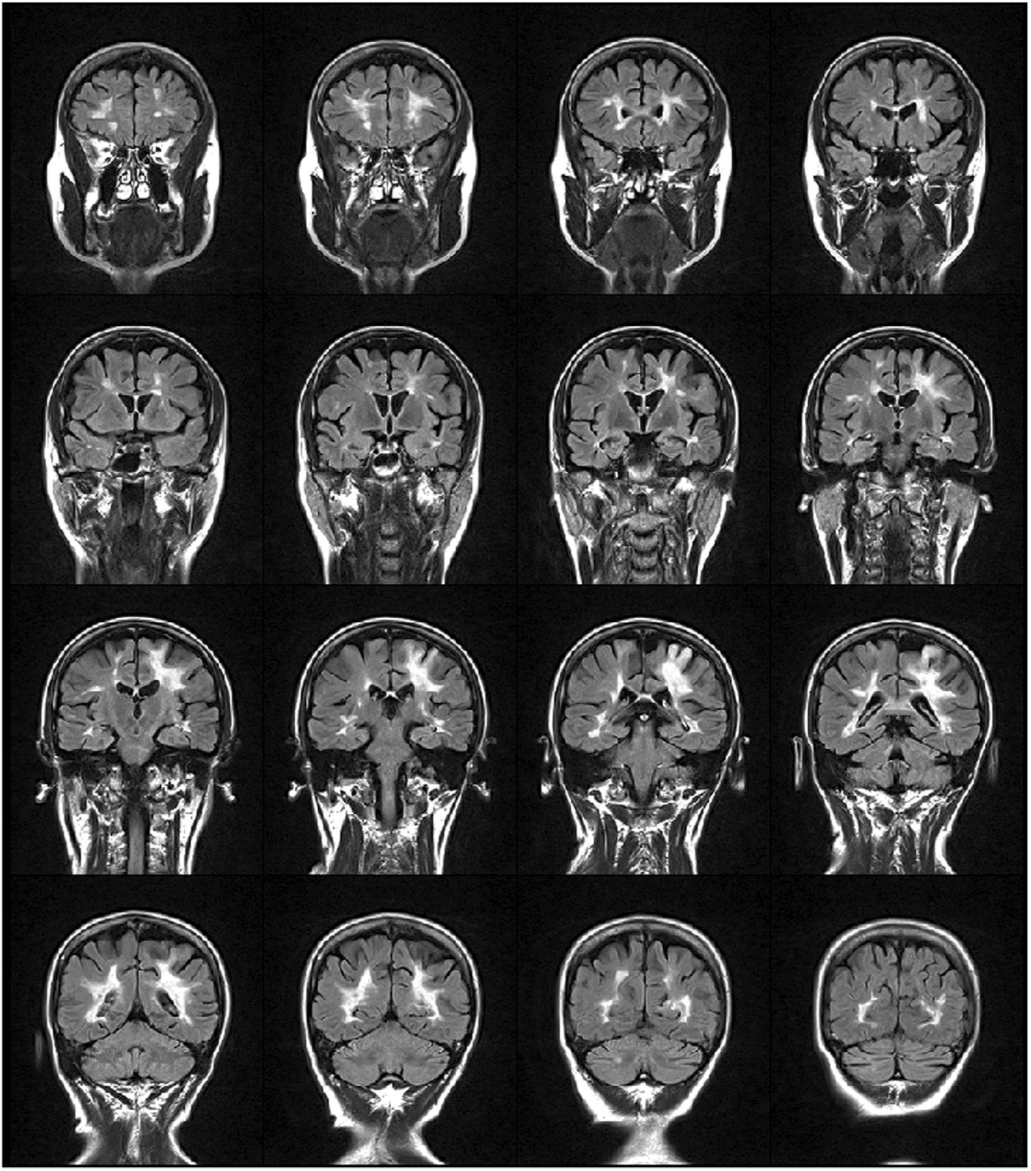
FLAIR weighted image showing multiple sclerosis lesions distributed mainly periventricularly and a hyperintense lesion in the fronto-parietal region that extends to the cortex, suggestive of progressive multifocal leukoencephalopathy.

We studied a 48-year-old right-handed female, diagnosed with relapsing-remitting MS in 2000. She received interferon beta-1a (Rebif 22^®^ and Rebif 44^®^) as immunomodulatory therapy from 2001 to 2010 with suboptimal adherence (frequent self-established drug holidays). In June 2012, the patient started treatment with monthly intravenous infusions of 300 mg natalizumab due to increased disease activity, following informed consent and according to the European Medicine Agency (EMA) (12). Subsequently, no clinical nor MRI signs of MS activity were evidenced until October 2013, when she presented a subacute motor deficit of the right UL, followed by involvement of the ipsilateral leg and urinary urgency. Neurological examination showed a moderate right hemiparesis with clumsiness of the hand and finger movement, hyperreflexia on four limbs, static and dynamic ataxia of the trunk and the right limbs. Cognition was normal. Her Expanded Disability Status Scale (EDSS) (13) score was 3.5. Brain MRI (Figure 1) showed a lesion in the left parietal lobe suggestive of acute PML (14). PML diagnosis was confirmed by cerebrospinal fluid analysis, positive for John Cunningham virus (JCV) presence (13 copies/ml). The patient was treated with 2 cycles of plasma exchange (PEX), without effects on the clinical ground. On follow-up MRI scans, performed between December 2013 and September 2014, immune reconstitution inflammatory syndrome did not occur nor the patient experience rebounding of MS-related disease activity. The patient complained, however, of a progressive worsening of motor impairment of the right UL. She became dependent in most activities of daily living (ADL), particularly she was unable to write and to ensure hygienic care. Consequently, an MDR treatment was prescribed by the referral neurologist following a consultation with our neurorehabilitation team.

When the patient was admitted to our inpatient rehabilitation unit (October 2014), neurological examination revealed a marked motor deficit and spasticity of the right UL, more pronounced at the hand and forearm, and a mild deficit and spasticity of the right leg. EDSS score was 5.5, Barthel Index (BI) (15) was 81/100. She suffered from moderate backache (pain intensity on visual analog scale—VAS—5/10). Clinical findings are reported in Table 1: moderate spasticity was found on the Modified Ashworth Scale (MAS) (16); the subject was able to perform only item 19 (hand to mouth) on the Action Research Arm Test shortened version (ARAT) (17). On the Canadian Occupational Performance Measure (COPM) (18), a client-centered instrument designed to identify occupational performance problems, patient had a low score for both performance and satisfaction domains.

**Table 1:** Patient performance on clinical and functional scales before and after MDR treatment.

		Before MDR	After MDR
EDSS		5.5	4.5
Barthel Index		81/100	89/100
Right UL MAS	Range: 0–4		
Biceps		2	1
Triceps		2	2
Wrist extensors		2	2
Wrist flexors		3	2
Fingers flexors		3	2
ARAT (item 19, “hand to mouth”), s		3.32	3.35
COPM-P	Range: 1–10	2.4	5.8
COPM-S	Range: 1–10	3.8	8.0

EDSS, Expanded Disability Status Scale; MAS, Modified Ashworth Scale; ARAT, Action Research Arm Test; COPM-P, Canadian Occupational Performance Measure-Performance; COPM-S, Canadian Occupational Performance Measure-Satisfaction

The patient underwent intensive MDR (5 days/week), with the main aim to improve right UL functions (i.e., the ability to grasp and to manipulate objects), to reduce spasticity and increase muscle strength.

Collectively, the MDR program included 23 sessions of neuromotor treatments (including functional electrical stimulation), 10 sessions of massages, 6 sessions of occupational therapy (OT). Contextually, treatments for lower limbs were delivered to improve dynamic balance and to improve control of the right foot during locomotion.

Treatments for UL consisted of passive mobilization of fingers, wrist, and elbow flexors to improve joint range of motion with the aim of increasing the patient’s ability in reaching and grasping. A customized brace was developed to keep the hand and wrist in a functional position. Functional electrical stimulation was applied to improve patient’s ability to voluntary activated fingers and wrist extensor (19) together with task-oriented rehabilitation and OT, using affordance and guiding techniques.

During the last part of the treatment period constraint-induced movement therapy was used (20, 21) to facilitate the use of the UL into ADL.

**Figure 2:**
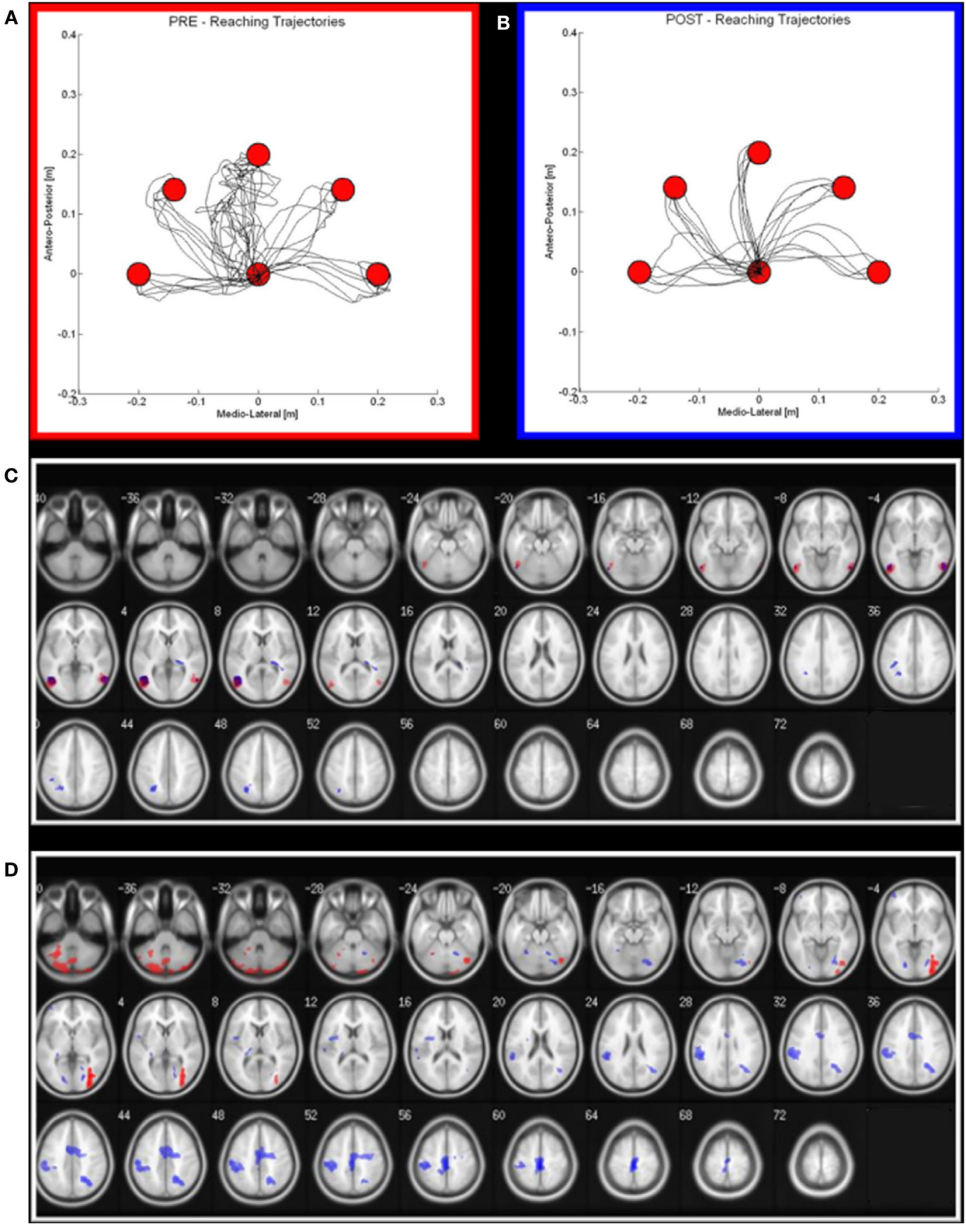
Robot output and functional MRI activation in pre (red) and post (blue) rehabilitation period. **(A,B)** on the left (red squared), the reaching trajectories before the robot-assisted rehabilitation; on the right (blue squared), the same trajectories after a 3-week period of robot-therapy. **(C,D)** Maxima of regions showing significant activations during the Observational task **(C)** and motor task **(D)** in pre—red areas—and post—blue areas—conditions. Transverse slice view (on a 152 T1 xjview template) of significant positive activation (thresholded at P FWE corrected <0.05 with *k* > 50 voxels).

Together with these treatments, the patient underwent daily sessions of robot therapy with a device called “Braccio di Ferro” (22). In details, the protocol consisted of a task-oriented rehabilitation approach that focuses on the practice of skilled motor performance (23, 24). The subject was seated on a chair, while grasping the handle of the robot with the treated hand. A large computer screen was used to display the current position of the hand and the target represented by circles. The task was center-out reaching movement, from one central target to five peripheral targets arranged on a semi-circle with a 20 cm radius (Figure 2A). After comparison of the target, no assistive force was delivered for a period of 2 s. At that time, unless the subject was able to reach the target on her own, a minimally assistive force was generated by the robot. This force was modulated according to the hand speed: robotic assistance was decreased if the hand speed grew, while it was increased if the hand speed decreased (24).

A t-test analysis was performed to compare assistive force, reaching duration, and number of sub-movements to reach the target (a measure of trajectory’s smoothness) before and after robot-therapy.

At the beginning and at the end of the rehabilitation period, the patient also underwent task-based fMRI, using blood oxygenation level dependent (BOLD) contrast using a 1.5 T scanner (Siemens Magnetom Avanto, Erlangen, Germany) with an 8-channel head coil.

A 3-dimensional T1-weighted magnetization prepared rapid gradient echo (TR/TE = 1,900/3.37 ms, FOV = 192 mm × 256 mm, in-plane resolution 1 mm × 1 mm, slice thickness = 1 mm, number of axial slices = 176) was acquired to be used as anatomical reference for fMRI analysis. Functional images were collected by gradient echo-planar (EPI) T2* sequence (TR = 3,000 ms; TE = 50 ms; flip angle = 90°; FOV = 180 mm; matrix size = 128 × 128; number of slices = 38; thickness = 4 mm) using BOLD contrast. Each fMRI session included four runs of 122 scans each (10 dummy scans). An AB block-design experimental paradigm was used as described elsewhere (25). Briefly, the paradigm consisted of 2 runs: in the first run, she was asked to observe (observation—O—run) and in the second run to execute hand grasping movements (motor—M—run). In the O run, the patient viewed movies of a hand grasping different objects. In the M run, the patient observed visual stimuli consisting in objects oriented in order to be grasped with the hand; she was asked to perform the grasping movement appropriate with respect to the shape of the object. In each run, during rest blocks, the subject observed still images of the effector just seen in O or M condition. Before the experiment, the patient was verbally instructed to execute the movements as if the object were close to her hand without reaching it. She was told to carry out the task only with the hand and the wrist and to repeat this action until the appearance of the next picture. She was also instructed to keep her gaze on the fixation point for the entire duration of the experiment, and to execute grasping movements about once every 2 s. The patient was able to perform the task in both conditions.

We used an MR-compatible visual system to present the stimuli (VisuaStim Digital system from Resonance Technology Inc.) and the use of E-Prime software (E-Prime 2.0 Psychology Software tool^1^) ensured exact timing of prompts during MR acquisitions. The subject performance in doing the task was visually assessed. fMRI data were analyzed using SPM12 (Wellcome Dept. Cogn. Neurol., London^2^). We modeled the expected hemodynamic response function with a block design. The six parameters describing head movements (three translations and three rotations) during scanning were included in the analysis as regressors of no interest.

We estimated four *t*-contrasts for the O condition: observation of a hand grasping at the pre-treatment (PreO), observation of a hand grasping at the post-treatment (PostO), observation of a hand grasping at the pre-treatment compared with observation of a hand grasping at the post-treatment (PreO vs PostO), observation of a hand grasping at the post-treatment compared with observation of an hand grasping at the pre-treatment (PostO vs PreO). The same four contrasts were estimated for the M condition (PreM; PostM; PreM vs PostM; PostM vs PreM). *T*-Contrasts tested for condition-related activation (*p*-corr < 0.05, corrected for multiple comparisons, Family Wise Error). When reporting results at the corrected level, only regions including 50 or more adjacent activated voxels were considered as statistically significant.

On discharge, after 40 days, the patient reported a reduction of right hand clumsiness, while hand extension, shoulder mobility, elbow flexion-extension, as well as static and dynamic postural stability, were all increased. Backache severity was decreased (VAS 3/10), while both BI and EDSS score changes indicated a functional improvement. An improvement in COPM performance and satisfaction score was also found (Table 1).

As regards the patient’s performance for robot-assisted reaching arm movements, at the end of the rehabilitation cycle, we observed an improvement of all the parameters (hereafter reported as mean values ± SDs at the beginning and at the end of the treatment, respectively), i.e., a significant reduction of the robot-generated assistive force (1.40 ± 1.82 N vs 0.24 ± 0.57 N; *p* < 0.01), of the reaching duration (4.45 ± 5.41 s vs 2.02 ± 0.27 s; *p* < 0.05) and of the number of sub-movements to reach the target (6.80 ± 8.35 vs 2.70 ± 0.99; *p* < 0.01), the latter suggesting an increase of movement’s smoothness.

At the end of the MDR treatment, several clusters of fMRI activation showed significant changes. Brain regions with significant activation in the Pre vs Post MDR are summarized in Table 2 and shown in Figure 2. In the PreM vs PostM contrast, increased activation was found bilaterally in the occipital visual areas (BA 17, 18, 19) and in the cerebellum. In the PostM vs PreM contrast, increased activation was seen in the right occipital visual areas (BA 18, 19), the right middle temporal gyrus (BA 21, 39), the temporal pole (BA 38) and in a sensory-motor network including the right supramarginal gyrus, the right inferior parietal lobule (BA 40), the right postcentral and the precentral gyrus (BA 6), the right medial frontal gyrus (BA 10), the left inferior frontal gyrus (BA 45) and the left putamen. In the PreO vs PostO and PostO vs PreO contrasts, fMRI analysis showed no supra-threshold clusters.

**Table 2:** MNI coordinate and *Z*-scores of the areas activated both during different task conditions, before and after MDR (BA, Brodmann area; L, left hemisphere; R, right hemisphere).

	PRE vs POST MDR								POST vs PRE MDR							
	Cluster size	Peak MNI coordinate			Side	Brain region	BA	*z* Value	Cluster size	Peak MNI coordinate			Side	Brain region	BA	*z* Value
	*k*	*x*	*y*	*z*					*k*	*x*	*y*	*z*				
Run 1: movement vs rest	208	−8	−90	8	L	Calcarine/cuneus	18/17	8.01	24,951	24	−78	32	R	Superior/middle occipital gyrus	18/19	11.81
		−2	−102	2	L	Calcarine/cuneus		5.70		44	−62	18	R	Middle temporal gyrus	39	11.63
	620	32	−88	0	R	Middle occipital gyrus	18	10.27		54	−58	30	R	Supramarginal gyrus/inferior parietal lobule	40	11.00
		34	−64	0	R	Middle occipital gyrus		8.83		46	−48	50	R	Postcentral gyrus		10.09
		34	−76	0	R	Inferior/middle occipital gyrus	18/19	8.20		24	−14	46	R	Precentral gyrus	6	9.24
									211	34	6	−36	R	Middle temporal gyrus/temporal pole	21/38	7.91
									1,333	16	52	−4	R	Medial frontal gyrus	10	5.93
										12	36	32	R	Anterior cingulate	32	5.90
									463	−34	24	6	L	Inferior frontal gyrus	45	5.72
										−24	12	0	L	Putamen		6.14
	489	−26	−80	−38	L	Cerebellum posterior lobe		6.69								
	309	−42	−60	−46	L	Cerebellum posterior lobe		7.05								
	363	50	−78	−34	R	Cerebellum posterior lobe		7.72								

Run 2: observation vs rest	No suprathreshold clusters								No suprathreshold clusters							

*Only regions that survived after FWE correction for multiple comparisons (*p*-corrected < 0.05, with *k* > 50 voxels) have been considered. See text for further details*.

According to the recommendations of the Declaration of Helsinki about ethical principles for medical research involving human subjects, both local ethics committee approval of the Don Gnocchi ONLUS Foundation and written informed consent from the subject to participate in the study and for the publication of this case report were obtained.

## Discussion

Our findings suggest that intensive MDR, including robot-assisted UL training, can be clinically effective in MS, even when the disease is complicated by superimposed PML. At the end of the treatment period, the patient showed a recovery of UL functions associated with better performances, despite the persistence of grasping deficits. We can, therefore, conclude that the clinical outcome of PML was ameliorated by rehabilitation, even if the treatment was administered a few months after the onset of this complication. It is widely accepted that, although PML is already known to be associated with better outcomes in MS compared to HIV, it may be further improved by *ad hoc* work-up, such as PEX or steroid treatment (4, 26). This was not the case for our patient, who did not show any benefit from PEX, but rather experienced a progressive worsening of the UL deficits provoked by PML, which was counteracted only by MDR.

We believe that the outcome of this case also confirms that robot-assisted UL treatment can be as effective in MS as in stroke rehabilitation (27) and that its association with OT can lead to an improvement in ADL. Moreover, the subject had severe UL involvement hampering the execution of other UE functional tests [such as 9-Hole Peg Test (28) or Box and Block Test (29) with the right UL], and we cannot provide any clinical follow-up data on a medium—to long-term ground, but the results we obtained are encouraging and further studies are warranted.

Many aspects of brain plasticity still remain to be elucidated in the MS rehabilitation field (9). Few longitudinal fMRI studies have demonstrated the usefulness of fMRI to monitor response to rehabilitation interventions (9, 11, 30–34).

The increased activation we found in the motor condition at the end of the rehabilitation period sustains the hypothesis that MDR has a significant impact on brain functional organization. Indeed, the areas showing significant change are all involved in motor performance, and it can be speculated that these findings reflect the restoration of function in underactive parts of the sensory-motor network. The changes we observed reinforce the idea that rehabilitation-induced plasticity is specifically linked to the trained function (motor abilities), and it is not merely a general effect on networks affected by the disease. However, the patient participation to a MDR treatment makes it difficult to disentangle the role played by its different components (neuromotor and robot therapies, gait training, OT) in driving the observed fMRI changes. Unfortunately, the lack of follow-up data does not allow to draw firm conclusions on the persistence of these changes, as well as on their impact on the clinical disease evolution.

Notwithstanding these limitations, this is the first evidence that MDR might still be helpful in cases of “dual” brain damage, such as PML superimposed to MS, indicating that, when PML provokes additional tissue damage in the MS brain, brain plasticity mechanisms remain several months after the index-event.

In conclusion, although the results from this case report are highly encouraging, additional studies are needed to confirm our findings. Larger samples of patients and with medium—to long-term follow-ups should be studied, with an additional aim of identifying potential clinical and paraclinical predictors of rehabilitation outcome.

## Ethics Statement

The study was approved by the Ethics Committee of Don Gnocchi Foundation, and informed written consent was obtained from the subject.

## Author Contributions

EG, FB, and DC: (1) data acquisition, analysis, interpretation, (2) drafting intellectual content, (3) final approval, and (4) responsibility for content. ET: (1) data acquisition, (2) drafting intellectual content, and (3) responsibility for content. NB: (1) data analysis and (2) responsibility for content. ST: (1) data acquisition, analysis, interpretation, (2) drafting intellectual content, and (3) responsibility for content. RP, IC, CG, and RC: (1) data acquisition and (2) responsibility for content. MR: (1) data analysis and interpretation, (2) drafting intellectual content, (3) final approval, and (4) responsibility for content.

## Conflict of Interest Statement

The authors declare that the research was conducted in the absence of any commercial or financial relationships that could be construed as a potential conflict of interest.

## Footnotes

^1^http://www.pstnet.com.

^2^http://www.fil.ion.ucl.ac.uk/spm.
